# Case Report: Six infants with primary intestinal lymphangiectasia: clinical manifestations of refractory cases and preliminary stratified clinical management experience

**DOI:** 10.3389/fped.2026.1949058

**Published:** 2026-09-16

**Authors:** Meng Shi, Ning Xue, Yunping Tang, Yumeng Zhang, Yuexia Bai, Xuxia Wei

**Affiliations:** 1 Department of Gastroenterology, Children's Hospital Affiliated to Shandong University (Jinan Children's Hospital), Jinan, China; 2 Department of Pathology, Children's Hospital Affiliated to Shandong University (Jinan Children's Hospital), Jinan, China

**Keywords:** case series, infant, PIL, refractory cases, serous cavity effusion, stratified clinical management

## Abstract

**Objective:**

This case series collected clinical data of six biopsy-confirmed infants with primary intestinal lymphangiectasia(PIL), summarized distinct clinical manifestations of refractory cases, and shared preliminary experience of risk-stratified clinical management for reference in rare pediatric gastrointestinal practice.

**Methods:**

We retrospectively enrolled six consecutive infants diagnosed with PIL via gastroscopic mucosal biopsy between January 2021 and June 2024. Patients were categorized into a favorable-response group (4 cases) and a refractory group (2 cases) according to clinical outcomes after 4 weeks of standardized exclusive medium-chain triglyceride (MCT) formula therapy. All clinical manifestations, laboratory indices, endoscopic and pathological findings, and long-term follow-up data were descriptively summarized without intergroup statistical comparisons.

**Results:**

All six infants presented with chronic watery diarrhea and generalized pitting edema; serous cavity effusion was detected in four patients, and hypocalcemic convulsions occurred only in the two refractory cases. Hypoalbuminemia and peripheral lymphocytopenia were universal laboratory abnormalities. Serum immunoglobulin G (IgG) levels could be falsely normal in patients receiving pre-admission intravenous immunoglobulin, which cannot exclude PIL diagnosis. Two endoscopic subtypes (edematous and granular) were identified. Pathological lymphatic dilation extending to the submucosa was exclusively observed in refractory infants. The combination of serous cavity effusion, serum albumin < 20 g/L, and hypocalcemic convulsions was only present in the two patients with treatment failure. One refractory child required long-term intermittent intravenous protein supplementation, while the other died of multiple organ dysfunction 3 months after admission.

**Conclusions:**

Clinical manifestations vary greatly among infants with PIL. The concurrent occurrence of serous cavity effusion, serum albumin < 20 g/L and hypocalcemic convulsions is a prominent clinical feature unique to refractory cases in this small sample. We shared preliminary stratified clinical management experience based on our single-center observation, and further large-sample multicenter studies are needed to verify our findings.

## Introduction

Primary intestinal lymphangiectasia (PIL, Waldmann disease) is a rare orphan protein-losing enteropathy characterized by diffuse dilatation of intestinal lymphatic vessels, leading to massive loss of protein-rich lymph into the intestinal lumen (1). Onset in infancy often presents non-specific clinical manifestations accompanied by severe complications including generalized anasarca, serous cavity effusion, and hypocalcemic convulsions, which delays definitive diagnosis and elevates mortality risk (2). Exclusive medium-chain triglyceride (MCT) formula serves as the first-line core therapy, as medium-chain lipids bypass intestinal lymphatic circulation and are directly absorbed via the portal vein to reduce lymphatic overload (3).

Marked inter-individual heterogeneity exists in treatment responses to standardized MCT dietary intervention. A subset of infants progresses to refractory disease with recurrent severe infections, intractable malnutrition, or fatal multi-organ dysfunction despite strict dietary compliance (4). Most existing publications on infantile PIL consist of isolated single-case reports. A previous large Chinese cohort summarized general epidemiological characteristics but failed to identify combined clinical markers to distinguish refractory patients from those with favorable outcomes (5). Reliable simple bedside screening indicators for high-risk infants remain absent in routine clinical practice.

This hypothesis-generating single-center retrospective case series included six infants with biopsy-verified PIL. We systematically compared clinical, laboratory, endoscopic, and pathological features between favorable and refractory subgroups, summarized a clinical triad-comprising serous cavity effusion, serum albumin < 20 g/L, and hypocalcemic convulsions-exclusive to treatment-failure infants, and put forward preliminary stratified clinical management experience for clinical reference. All conclusions derived from this limited sample are observational and require further verification in larger cohorts.

## Case presentation

### Enrollment criteria and clinical definitions

#### Inclusion & exclusion criteria

We screened all infant patients admitted to the Department of Gastroenterology, Jinan Children's Hospital between January 2021 and June 2024, and enrolled infants with symptom onset before 12 months of age, definite PIL diagnosis confirmed by gastroscopic mucosal biopsy consistent with the French National Diagnostic Protocol (1) and the 8th edition of Zhu Futang Textbook of Pediatrics (Chinese textbook) (6), and complete clinical, laboratory, endoscopic, pathological and follow-up data; patients with secondary intestinal lymphangiectasia induced by sepsis, inflammatory bowel disease, tuberculosis, cardiac disorders or malignancy, incomplete follow-up records, suspected CHAPLE disease or severe congenital gastrointestinal malformations were excluded. Eight infants with suspected PIL were initially screened; two cases were excluded (one secondary to neonatal sepsis, one lost to follow-up), leaving six eligible patients with complete long-term follow-up. Clinical data were independently extracted by two pediatric gastroenterologists, with discrepancies resolved via senior physician group discussion.

All six enrolled patients were diagnosed by gastroduodenal biopsy via gastroscopy. Patients diagnosed without biopsy confirmation were excluded, which may introduce selection bias and limit the generalizability of our findings.

## Standardized definition of serous cavity effusion

Serous cavity effusion was defined as detectable fluid collection in the peritoneal, pleural, or pericardial space confirmed by abdominal ultrasound, chest radiography, or cardiac echocardiography. This unified term covers fluid involvement of a single cavity or multiple cavities. Routine thoracic ultrasound was not performed in early enrolled patients, which may lead to underestimation of mild pleural fluid. The presence of serous cavity effusion indicates decompensated intestinal lymphatic leakage and severe systemic protein loss, regardless of the number of involved serous compartments (7).

### Isolated ascites

serous cavity effusion limited to the peritoneal cavity only;

### Multi-compartment serous cavity effusion

fluid accumulation in ≥2 serous spaces.

## Treatment response stratification

Patients were divided into two groups based on clinical performance after 4 weeks of standardized exclusive MCT formula feeding:

### Favorable outcome group

Obvious relief of diarrhea and edema within 2 weeks, sustained elevation of serum albumin, and no long-term dependence on intermittent intravenous albumin or immunoglobulin replacement;

### Refractory group

Persistent unimproved symptoms after 4 weeks of dietary therapy, continuous demand for regular intravenous protein support, or progressive deterioration leading to death.

The 4-week cutoff was based on clinical observation: most responders show improvement within 1–2 weeks of MCT therapy. This definition is operational and is not derived from an established guideline.

## Baseline clinical characteristics of the six infants

The cohort included 2 males and 4 females, with symptom onset ranging from 2.6 to 6.5 months (mean 3.9 ± 1.3 months). The initial chief complaint of all patients was chronic watery diarrhea and symmetric generalized pitting edema. Serous cavity effusion was identified in 4/6 (66.7%) infants, while hypocalcemic convulsions only occurred in the two refractory patients. Universal laboratory abnormalities included hypoalbuminemia and peripheral lymphocytopenia. These characteristics are summarized in Table 1.

**Table 1:** General characteristics and treatment response grouping of six infants with PIL, including sex, age at onset, clinical manifestations, serous cavity effusion, hypocalcemic convulsions, and treatment response.

Case	Sex	Age at onset(mo)	Main clinical manifestations	Serous cavity effusion	Hypocalcemic convulsions	Treatment response
1	F	3.0	Diarrhea, bilateral lower limb edema	None	No	Favorable
2	M	2.6	Diarrhea, facial and bilateral lower limb edema	None	No	Favorable
3	F	5.0	Diarrhea, bilateral lower limb edema	Ascites(isolated)	No	Favorable
4	F	6.5	Diarrhea, scrotal and bilateral lower limb edema	Ascites(isolated)	No	Favorable
5	M	4	Diarrhea, generalized edema	Ascites (isolated)	Yes	Poor
6	F	3	Diarrhea, generalized edema	Ascites, pleural effusion，pericardial effusion	Yes	Poor
Summary	2M/4F	2.6–6.5 (3.9 ± 1.3)	-	4/6 (66.7%)	2/6 (33.3%)	Favorable:4; Poor:2

Continuous variables are expressed as mean ± standard deviation (SD) with range; categorical data are presented as *n* (%). mo, month. Isolated ascites refers to serous cavity effusion limited solely to the peritoneal cavity without pleural or pericardial fluid accumulation.

## Favorable outcome group (cases 1–4, *n* = 4)

All four infants achieved rapid symptom relief within 1–2 weeks of MCT initiation. Two patients presented with isolated ascites without hypocalcemic convulsions, and none required repeated long-term intravenous protein transfusion. Complete resolution of clinical symptoms and age-appropriate catch-up growth were achieved during follow-up. Notably, Case 2 exhibited severe hypoalbuminemia (13.7 g/L) at admission, yet lacked serous cavity effusion and convulsive episodes, and attained full remission via dietary therapy alone (8). This finding indicates isolated severe hypoalbuminemia cannot predict refractory disease in the absence of the other two triad components.

## Refractory group (cases 5–6, *n* = 2)

Both refractory infants exhibited the complete clinical triad: serous cavity effusion, serum albumin < 20 g/L, and hypocalcemic convulsions.

Case 5 presented with isolated ascites and recurrent hypocalcemic twitching, with a minimum serum albumin of 17.2 g/L. Long-term outpatient intermittent albumin and immunoglobulin infusions were required to control persistent edema and recurrent infections despite strict MCT adherence.

Case 6 developed multi-compartment serous cavity effusion (ascites, pleural, and pericardial fluid) accompanied by severe generalized convulsions. The lowest serum calcium during convulsive episodes reached 1.02 mmol/L, with a nadir albumin level of 15.4 g/L (9). Comprehensive intensive therapy failed to halt disease progression, and the infant died of multiple organ dysfunction syndrome 3 months after admission.

It should be emphasized that this triad was observed only in these two cases within our small cohort. The predictive prognostic performance of this triad cannot be established based on the current sample size.

## Laboratory, endoscopic and pathological findings

### Laboratory indices

Hypoalbuminemia and peripheral lymphocytopenia were detected in all six patients. Four infants had reduced serum immunoglobulin G (IgG) concentrations, whereas two showed normal IgG levels due to pre-hospital exogenous immunoglobulin infusion. This critical clinical reminder indicates normal serum IgG cannot rule out PIL diagnosis if patients receive prior intravenous immunoglobulin; comprehensive clinical, endoscopic, and pathological evaluation is mandatory for definitive diagnosis (10).

The admission serum calcium value of Case 6 was within the normal range (2.00 mmol/L), while the nadir calcium measured during convulsions dropped to 1.02 mmol/L, highlighting the limitation of single-time-point biochemical testing and the necessity of dynamic monitoring for electrolyte disturbances. Only the two refractory patients met the albumin threshold of < 20 g/L, a core component of the clinical triad observed in refractory cases. Detailed laboratory parameters are presented in Table 2.

**Table 2:** Main laboratory findings and treatment response of six infants with PIL, including lymphocyte count, albumin, IgG, serum potassium, calcium, and magnesium.

Case	Lymphocyte count ( × 10⁹/L)	Albumin (g/L)	IgG(g/L)	Serum potassium (mmol/L)	Serum calcium (mmol/L)	Serum magnesium (mmol/L)	Treatment response
1	1.19	23.1	0.27	2.8	1.33	0.27	Favorable
2	0.93	13.7	2.70	3.6	1.84	0.40	Favorable
3	1.24	20.5	1.99	4.1	1.73	0.35	Favorable
4	0.50	34.1	4.69	3.5	2.20	0.80	Favorable
5	0.25	18.9	0.42	2.9	0.74	0.31	Poor
6	0.92	15.4	0.17	2.5	2.00*	0.26	Poor

The admission serum calcium value of Case 6 was 2.00 mmol/L, while the minimum calcium concentration measured during generalized convulsive episodes reached 1.02 mmol/L. Case 5 developed hypocalcemic convulsions with a calcium nadir of 0.74 mmol/L. Serum albumin < 20 g/L constitutes one core component of the clinical triad observed in refractory PIL cases.

## Endoscopic subtypes and pathological grading

All six patients underwent uncomplicated gastroscopy with mucosal biopsy. Two endoscopic subtypes were classified according to published standards (11):

Edematous type (3 cases): Swollen intestinal villi without characteristic white mucosal lesions;

Granular type (3 cases): Scattered rice-grain white patchy lesions on the duodenal mucosa (Figure 1).

**Figure 1:**
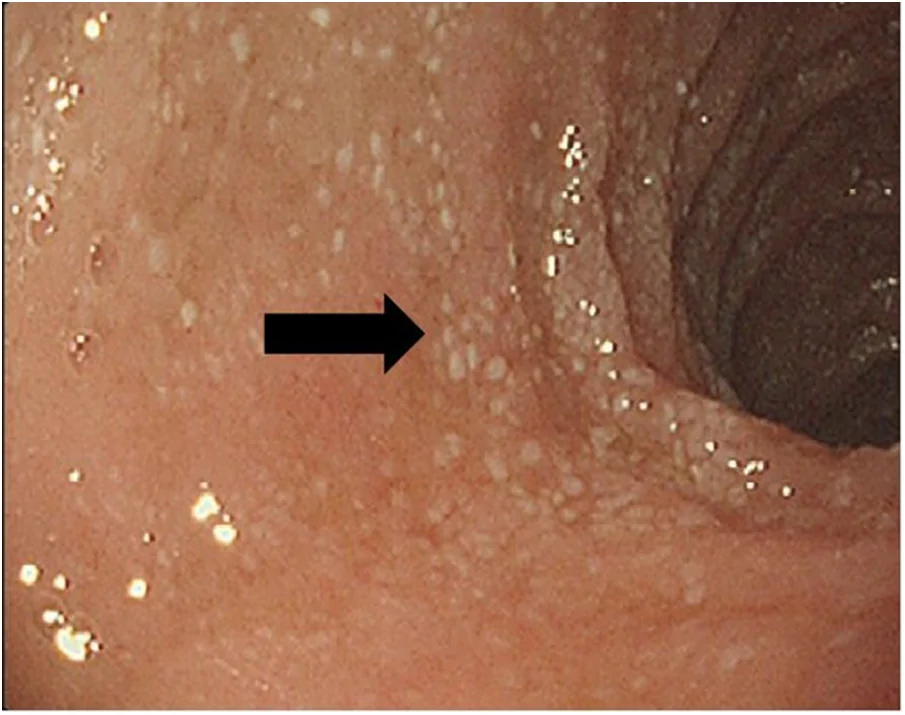
Representative endoscopic image of infantile PIL (granular type). Characteristic scattered/patchy white spots on the duodenal mucosa (arrows), resembling rice grains. This subtype corresponds to the granular type described by Meng et al. (11).

Pathological grading was defined by the depth of intestinal wall lymphatic dilation:

Mild: Lymphatic dilatation limited exclusively to the lamina propria;

Moderate: Lymphatic dilatation involving both lamina propria and submucosa;

Severe: Full-thickness dilatation extending to the muscular layer.

All four favorable-outcome patients presented mild pathological changes confined to the lamina propria (Figure 2). Both refractory infants exhibited moderate lesions with lymphatic expansion invading the submucosa, indicating more extensive systemic lymphatic damage.

**Figure 2:**
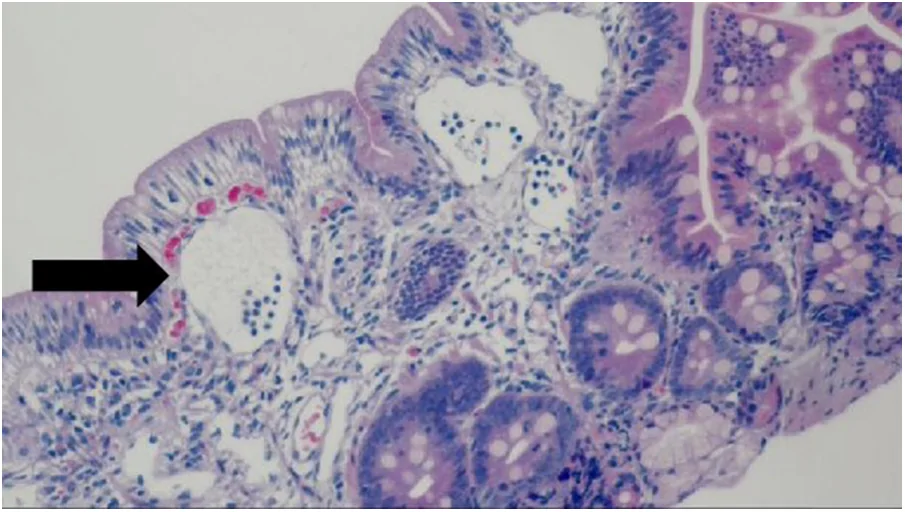
Representative histopathological image of mild infantile PIL (HE staining, original magnification   × 200). Dilated lymphatic vessels are visible in the lamina propria (arrows). According to the grading criteria by Meng et al. (11), lymphatic dilation confined to the lamina propria represents mild pathological severity. In our cohort, such mild changes were observed exclusively in the favorable prognosis group.

A detailed summary of endoscopic subtypes, corresponding pathological diagnoses, and pathological severity for each case is provided in Table 3.

**Table 3:** Gastroscopic findings and pathological results of six infants with PIL, including endoscopic subtype, pathological diagnosis, and severity grading (mild/moderate/severe).

Case	Gastroscopic findings	Pathological diagnosis (mucosal biopsy)	Pathological Severity
1	Rough mucosa in duodenal bulb, descending part, and jejunum; short and thick villi	Moderate to severe atrophy and tortuosity of duodenal villi; Mild lymphatic dilation confined to the lamina propria of several villi	Mild
2	Edematous mucosa from duodenal bulb to proximal jejunum	Lymphatic dilation confined to the lamina propria of duodenal and jejunal villi	Mild
3	Edematous and thickened villi in duodenal bulb, descending, horizontal, and ascending parts, and proximal jejunum; some rice grain-like changes	Mild lymphatic dilation confined to the lamina propria	Mild
4	Edema in duodenal bulb, descending part, and jejunum; coarse villi and diffusely distributed white spots	Lymphatic dilation confined to the lamina propria of villi	Mild
5	Smooth mucosa in duodenal bulb and descending part; rough mucosa in bulb and descending junction; edematous proximal jejunal mucosa with coarse villi	Lymphatic dilation involving lamina propria (mild) with focal extension to submucosa (moderate)	Moderate
6	Rough mucosa in duodenal bulb, descending, and horizontal parts; white spot-like changes	Lymphatic dilation involving lamina propria with extension to submucosa (moderate)	Moderate

Endoscopic subtypes and pathological severity grading criteria were adopted from Meng et al. (11). Granular type: intestinal mucosa with scattered rice-grain white lesions; Edematous type: swollen villi without white mucosal changes. Pathological grades: Mild = lymphatic dilation confined to lamina propria; Moderate = dilation involving lamina propria and submucosa; Severe = full-thickness dilation extending to the muscular layer.

## Representative case illustrations

### Case 2 (favorable phenotype: isolated severe hypoalbuminemia without the complete triad)

Case 2 (Favorable phenotype) presented with severe hypoalbuminemia (13.7 g/L) without serous cavity effusion or hypocalcemic convulsions, and achieved full remission with MCT diet alone, confirming that isolated severe hypoalbuminemia without the other two triad components carries minimal risk of refractory disease (Table 1, Table 2) (12).

### Case 6 (refractory high-risk phenotype with the full triad)

A 3-month-old male infant was hospitalized for intractable diarrhea, severe generalized edema, poor feeding, and intermittent limb twitching. Cross-sectional imaging confirmed multi-compartment serous cavity effusion; laboratory testing recorded a convulsive-phase calcium nadir of 1.02 mmol/L and albumin of 15.4 g/L (9). Granular-type mucosal lesions were observed endoscopically, with pathological confirmation of moderate lymphatic dilation extending to the submucosa.

Aggressive combined intervention including strict MCT feeding, repeated albumin/immunoglobulin transfusions, and continuous electrolyte correction failed to reverse progressive malnutrition and recurrent infections. Long-term unfavorable outcomes in PIL patients with extensive multi-compartment fluid accumulation have been previously documented (13), which aligns with the fatal progression observed in Case 6.

## General therapeutic regimen and long-term follow-up

All patients received exclusive high-MCT formula as first-line treatment, combined with standardized supportive care including electrolyte correction and regular supplementation of fat-soluble vitamins, zinc, and selenium to prevent secondary nutritional deficiencies (14). The median follow-up duration was 12 months (range: 6–24 months). All four favorable-response infants maintained sustained remission and achieved normal growth milestones. Among refractory cases, one required lifelong intermittent intravenous protein replacement, and the other experienced fatal disease progression.

## Discussion

Case series based on consecutive real-world patients are valuable hypothesis-generating tools for rare orphan diseases such as infantile PIL. This six-case single-center cohort confirmed prominent phenotypic heterogeneity in MCT treatment responses and identified a consistent combination of severe clinical manifestations exclusively present in refractory infants. The preliminary stratified clinical management experience summarized herein aims to provide simple bedside guidance for pediatric gastroenterologists, while all findings require external validation in larger multicenter cohorts (15).

### Mechanistic interpretation of the clinical triad seen in refractory cases

The two treatment-failure infants in our cohort uniformly manifested three concurrent features: serous cavity effusion, serum albumin < 20 g/L, and hypocalcemic convulsions. This combination serves as a bedside alert marker for high-risk refractory PIL, with a clear pathological basis supported by biopsy results:

Submucosal lymphatic damage causes massive lymph extravasation across serosal surfaces, leading to serous cavity effusion, a marker of impaired major lymphatic trunks such as the thoracic duct (7);

Sustained overwhelming intestinal protein loss exceeds hepatic synthetic compensation, resulting in decompensated severe hypoalbuminemia below 20 g/L (8);

Concurrent loss of calcium, magnesium, and vitamin D transport proteins induces refractory hypocalcemia and recurrent convulsive episodes (9).

Even isolated ascites accompanied by the other two triad components signals severe decompensated lymphatic leakage and a refractory clinical trajectory. We recommend routine thoracic ultrasound for newly diagnosed infants, as this modality detects subtle pleural fluid more sensitively than chest radiography and avoids missed identification of high-risk features (7).

### Key clinical diagnostic and differential insights

A major diagnostic pitfall observed in this cohort is falsely normalized serum IgG secondary to pre-admission intravenous immunoglobulin infusion. Clinicians must avoid relying solely on immunoglobulin levels to rule out PIL and integrate clinical symptoms, endoscopy, and histopathology for definitive diagnosis (10).

Refractory infants meeting the full triad require targeted screening for monogenic mimics of sporadic PIL, particularly CHAPLE disease (CD55 deficiency), a treatable protein-losing enteropathy responsive to complement inhibitor therapy (16). CD55 genetic testing was not performed in our cohort, so coexisting CHAPLE disease cannot be fully excluded, representing an important limitation of our retrospective case series.

Histologically, submucosal lymphatic dilatation was only detected in patients with the complete triad, providing histological evidence that this clinical combination reflects widespread systemic lymphatic dysfunction rather than localized intestinal leakage (11). Multidisciplinary consultation and early therapeutic escalation are strongly recommended once all three triad features are identified to optimize patient outcomes (17).

### Preliminary stratified clinical management experience

Combining published pediatric PIL guidelines and our single-center observational experience, we summarized a preliminary stratified clinical management workflow based on the presence of the triad (Figure 3):

**Figure 3:**
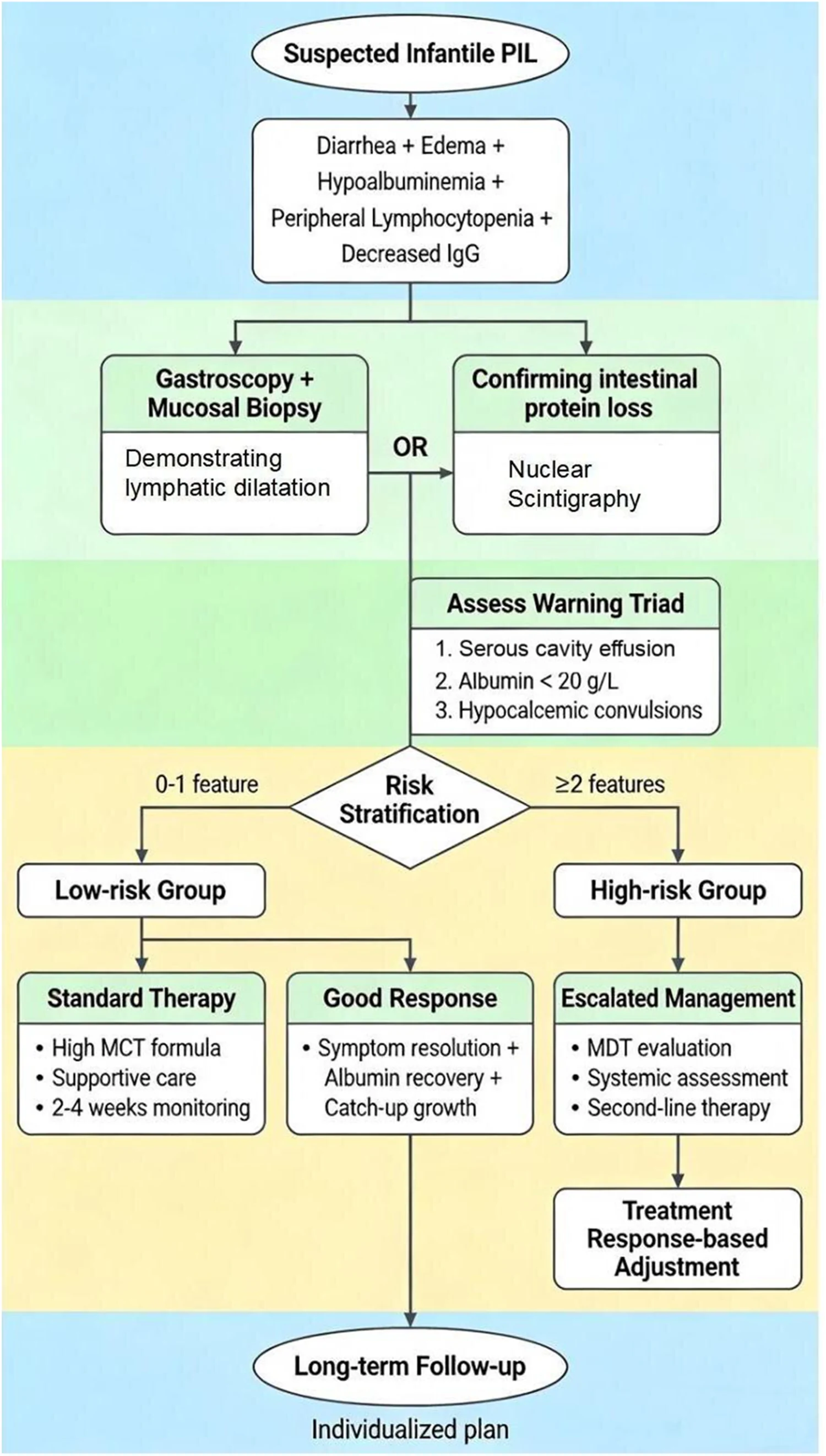
Preliminary stratified clinical management workflow for infantile PIL based on the clinical triad (serous cavity effusion, serum albumin < 20 g/L, and hypocalcemic convulsions). Low-risk infants (0-1 feature) receive exclusive MCT formula with supportive care and 2-4 weeks of monitoring. High-risk infants (≥2 features) require immediate MDT consultation, systemic lymphatic imaging, genetic evaluation, and consideration of second-line therapy. This workflow is for clinical research reference only and cannot replace individualized clinical decision-making.

Low-risk infants (0 or 1 triad component): Standard exclusive MCT formula plus comprehensive nutritional support with 2–4 weeks of close clinical monitoring. Most patients in this subgroup achieve sustained disease control with dietary therapy alone (12).

High-risk infants (≥2 triad components or complete triad): Immediate multidisciplinary team (MDT) consultation involving pediatric gastroenterology, clinical nutrition, radiology, and medical genetics. Systemic magnetic resonance lymphangiography is recommended to evaluate the full extent of lymphatic injury (10), alongside genetic screening for lymphatic dysplasia genes and CHAPLE disease (18).

For high-risk infants with persistent symptoms after 4 weeks of standardized MCT therapy, off-label sirolimus may be considered under strict Multidisciplinary team (MDT) supervision. Sirolimus inhibits lymphatic endothelial proliferation and reduces intestinal lymph leakage, with supportive evidence limited to small case series and individual reports (19, 20). In addition to the adverse effects already mentioned (myelosuppression, hepatotoxicity, hyperlipidemia), infant-specific risks should be carefully considered, including immunosuppression-related severe infections and potential impact on growth and development. Rigorous monitoring of blood counts, liver function, and serum lipids is mandatory during administration to screen for myelosuppression and hepatotoxicity. Full informed consent detailing limited clinical evidence must be obtained from caregivers prior to sirolimus initiation.

Long-term follow-up protocols should include regular measurement of zinc, selenium, and fat-soluble vitamins; selenium deficiency increases myocardial dysfunction risk, while vitamin D insufficiency exacerbates hypocalcemic convulsions (14).

Sandostatin has been reported in some case series as a therapeutic option for refractory PIL. However, to date, no dedicated case series has focused specifically on infantile PIL, and safety data in infants remain extremely limited, particularly regarding risks such as necrotizing enterocolitis and potential growth suppression. In our institutional practice, Sandostatin has not been included in the therapeutic armamentarium for infantile PIL. Given the extremely limited evidence and lack of prior institutional experience with Sandostatin in this age group, it was not included in the therapeutic decision-making for these two cases.

## Comparison with previously published PIL literature

A large Chinese single-center cohort of 42 infants and young children with PIL reported comparable rates of serous cavity effusion and convulsions but did not analyze whether combined clinical manifestations could stratify treatment response (5). Our case series extends prior epidemiological descriptions by identifying a distinct high-risk phenotype defined by three coexisting severe complications, answering the unmet clinical question of which infants require intensified therapy beyond standard MCT feeding.

Notably, infantile PIL presents far more life-threatening complications than adult-onset disease. Adult patients typically manifest insidious peripheral edema and fatigue, whereas extensive serous cavity effusion and hypocalcemic convulsions are rare in adult cohorts (3). This discrepancy stems from immature infant lymphatic development, higher metabolic demands, and limited organ functional reserve, highlighting the necessity of age-specific risk assessment criteria that cannot be extrapolated from adult clinical experience.

Notably, the Li et al. (5) cohort included patients up to 3 years of age and utilized various diagnostic modalities including enteroscopy and imaging, whereas our series was restricted to biopsy-proven infants <12 months. These differences in enrollment age and diagnostic criteria may contribute to the higher proportion of refractory cases (33.3%) observed in our cohort, as a younger age and stricter diagnostic criteria may select for a more severe disease spectrum.

## Limitations of the present case series

This work is a retrospective single-center case series limited to only six consecutive infant patients, with merely two refractory cases included. Infantile PIL is an ultra-rare orphan disorder; even after 3.5 years of continuous enrollment at a provincial tertiary children's hospital, sample size remains small, restricting the generalizability of our observations and summarized management experience. Due to the limited sample size, sensitivity, specificity, positive predictive value, negative predictive value, and other diagnostic performance metrics of the proposed triad cannot be calculated. All conclusions are hypothesis-generating and require validation in large-scale multicenter prospective cohorts (21).

Additional inherent limitations include:

Thoracic ultrasound was not routinely performed in early patients, potentially underestimating mild pleural serous cavity effusion;

CD55 and lymphatic dysplasia genetic testing were not completed for all six infants, precluding complete exclusion of monogenic mimics such as CHAPLE disease (16);

Fecal *α*1-antitrypsin clearance and nuclear scintigraphy were not uniformly implemented, creating mild diagnostic selection bias limited to gastroscopy-confirmed cases (21).

No subject in our cohort received sirolimus. Therefore, the recommendations for sirolimus use are derived from published literature and institutional experience rather than original clinical data from this study. Future prospective studies are needed to evaluate the efficacy and safety of sirolimus in this population.

The findings of this study are derived exclusively from biopsy-proven infants with PIL under 12 months of age. Therefore, these results should not be generalized to older children or to PIL patients without biopsy confirmation.

Future multicenter research should integrate genetic testing, non-invasive protein-losing screening, and systemic lymphatic imaging to establish a complete diagnostic and prognostic workflow for infantile PIL, with extended long-term monitoring of growth and neurodevelopmental trajectories in surviving refractory infants.

## Conclusion

Infants diagnosed with PIL exhibit heterogeneous clinical responses to standard MCT dietary therapy. The co-occurrence of serous cavity effusion, serum albumin < 20 g/L, and hypocalcemic convulsions constitutes a unique clinical feature only seen in refractory patients within this small single-center cohort. Clinicians should rapidly recognize this high-risk phenotype at initial admission, arrange timely multidisciplinary evaluation, and implement early therapeutic escalation to improve patient survival. The preliminary stratified clinical management experience summarized herein is derived solely from six observational infant cases and requires further verification in larger multicenter patient cohorts (15).

## Data Availability

The datasets presented in this article are not readily available because of ethical and privacy restrictions. Requests to access the datasets should be directed to the corresponding author.
